# Supplementary material for: Simultaneous Enhancement of Thermostability and Catalytic Activity of a Metagenome-Derived β-Glucosidase Using Directed Evolution for the Biosynthesis of Butyl Glucoside
Source: Int J Mol Sci. 2019 Dec 10;20(24):6224. doi: 10.3390/ijms20246224 (PMC6940790; doi:10.3390/ijms20246224)
Supplement: Supplementary file 1 [file ijms-20-06224-s001.pdf]

Supplementary Files

Title: Simultaneous enhancement of thermostability and catalytic activity of a metagenome-derived β-glucosidase with directed evolution for biosynthesis of butyl glucoside

Authors: Bangqiao Yin[1, †], Qinyan Hui[1, †], Muhammad Kashif[1], Ran Yu[1], Si Chen[1], Qian Ou[1], Bo Wu [1, 2], and Chengjian Jiang [1*]

Affiliations:

[1] State Key Laboratory for Conservation and Utilization of Subtropical Agro-bioresources, Guangxi Microorganism and Enzyme Research Center of Engineering Technology, College of Life Science and Technology, Guangxi University, Nanning 530004, China.

[2] Department of chemical and biological engineering, Guangxi Normal University for Nationalities, Chongzuo 532200, China.

[†] These authors contributed equally to this work.

[*]**Corresponding author:**

[*]E-mail: jiangcj0520@vip.163.com

[*]Tel: +86-771-3270736

Table S1 Substrate specificity of Bgl1D2, Bgl1D6, and Bgl1D20 enzymes.

Substrate	Linkage of glycosyl group	Specific activity(U/mg)				
		Bgl1D2	Bgl1D6	Bgl1D20	Y82S	W122G
Aryl-glycosides						
ρNP-β-D- glucopyranoside	βGlc	26.43 ± 0.17	16.67 ± 0.11	235.88 ± 0.32	-[c]	-[c]
ρNP-β-D-galactopyranoside	βGal	16.78 ± 0.03	ND[a]	15.37 ± 4.21	14.37± 0.43	ND[a]
oNP-β-D-galactopyranoside	βGal	8.28 ± 0.12	ND[a]	6.42 ± 0.22	7.84±0.21	ND[a]
ρNP-α-D-glucopyranoside	αGlc	ND[a]	ND[a]	ND[a]	-[c]	-[c]
ρNP-N-acety-β-D-glucosaminide	βGlc	0.23 ± 0.03	0.11 ± 0.02	1.63 ± 0.46	-[c]	-[c]
ρNP-β-D-xylopyranoside	βXyl	ND[a]	ND[a]	ND[a]	-[c]	-[c]
Salicin	βGlc	1.45 ± 0.29	0.99 ± 0.10	29.49 ± 4.25	-[c]	-[c]
Saccharides					-[c]	-[c]
Sophorose	Glcβ(1,2)Glc	ND[a]	ND[a]	ND[a]	-[c]	--[c]
Cellobiose	Glcβ(1,4)Glc	56.94 ± 0.44	99.58 ± 0.10	181.57 ±3.69	-[c]	-[c]
Lactose	Galβ(1,4)Glc	32.04 ± 2.10	ND[a]	25.32 ± 5.28	30.24± 0.82	ND[a]
Trehalose	Glcα(1,1)Glc	ND[a]	ND[a]	ND[a]	-[c]	-[c]
Maltose	Glcα(1,4)Glc	ND[a]	ND[a]	ND[a]	-[c]	-[c]
Isomaltose	Glcα(1,6)Glc	ND[a]	ND[a]	ND[a]		-[c]
Mannose	Glcα(1,4)Glc	ND[a]	ND[a]	ND[a]	-[c]	-[c]
Sucrose	Glcα(1,2)Fru	ND[a]	ND[a]	ND[a]	-[c]	-[c]
Xylan	βXyl	10.05 ± 0.50	8.98 ± 0.31	9.43 ± 3.15	-[c]	-[c]
CMC	βGlc	5.12 ± 0.61	5.89 ± 0.40	15.22 ± 4.83	-[c]	-[c]
Souble starch	αGlc	ND[a]	ND[a]	ND[a]	-[c]	-[c]
Starch from wheat	αGlc	ND[a]	ND[a]	ND[a]	-[c]	-[c]
4-Methylumbelliferyl-β-D-glucopyranoside	βGlc	-[b]	-[b]	-[b]	-[c]	-[c]

ND[a]: Not detected

-[b]: Fluorescence can be detected

-[c]: unknown

Table S2 Effect of various organic solvents on enzyme activity of Bgl1D2.

Organic solvent	Relative activity (%)				
Bgl1D2	Concentration (%)				
	10	20	30	40	50
None	100.00±0.09	100.00±0.31	100.00±0.07	100.00±0.20	100.00±0.16
Methanol	97.84±0.06	69.10±0.19	23.53±0.75	4.00±0.80	3.46 ±0.09
Ethanol	98.20±0.26	98.61±0.46	93.30±0.44	15.72±0.10	10.85±0.10
Octanol	105.03±0.22	103.48±0.31	108.03±0.46	104.10±0.08	109.08±0.06
I-Propanol	96.91±0.23	95.37±0.58	96.89±0.15	16.68±0.19	16.76 ±0.68
Isopropyl alcohol	96.31±0.43	96.25±0.07	96.71±0.75	93.84±0.42	95.56±0.07
Glycerol	100.53±0.08	98.02±0.26	38.23±0.22	11.22±0.61	11.46±0.51
Butanol	103.72±0.72	98.79±0.51	94.78±0.27	94.78±0.51	86.48 ±0.13
Acetonitrile	95.49±0.23	0.55±0.06	0.83±0.29	0.27±0.10	0.22 ±0.33
Acetone	76.99±0.83	22.72±0.40	11.97±0.22	2.72±0.34	1.55 ±0.80
Ethyl acetate	98.85±0.61	85.85±0.54	87.29±0.69	69.99±0.97	60.54±0.04

Table S3 Effect of various organic solvents on enzyme activity of Bgl1D6.

Organic solvent	Relative activity (%)				
Bgl1D6	Concentration (%)				
	10	20	30	40	50
None	100.00±0.00	100.00±0.42	100.00±0.04	100.00±0.04	100.00±0.10
Methanol	97.37±0.12	80.28±0.35	39.02±0.84	12.05±0.08	1.75±0.40
Ethanol	80.26±0.70	68.98±0.14	47.26±0.88	43.38±0.08	14.06±0.35
Octanol	101.24±0.11	103.02±0.66	101.97±0.22	102.75±0.21	105.29±0.10
I-Propanol	19.74±0.96	18.43±0.14	14.73±0.66	12.61±0.36	2.32±0.29
Isopropyl alcohol	56.67±0.59	51.46±0.44	24.14±0，07	8.73±0.71	3.40±0.49
Glycerol	100.09±0.44	101.51±0.13	101.40±0.59	94.26±0.43	95.20±0.66
Butanol	37.93±0.64	41.60±0.09	85.04±0.33	97.78±0.22	91.01±0.32
Acetonitrile	11.26±0.68	6.06±0.95	4.22±0.84	0.47±0.18	1.97±0.49
Acetone	59.53±0.14	49.83±0.28	12.85±0.17	11.47±0.95	1.58±0.41
Ethyl acetate	101.30±0.18	95.16±0.29	94.36±0.16	96.15±0.38	82.65±0.85

Table S4 Effect of various organic solvents on enzyme activity of Bgl1D20.

Organic solvent Bgl1D20	Relative activity (%) Concentration (%)				
	10	20	30	40	50
None	100.00±0.00	100.00±0.42	100.00±0.04	100.00±0.04	100.00±0.10
Methanol	97.37±0.12	80.28±0.35	39.02±0.84	12.05±0.08	1.75±0.40
Ethanol	80.26±0.70	68.98±0.14	47.26±0.88	43.38±0.08	14.06±0.35
Octanol	101.24±0.11	103.02±0.66	101.97±0.22	102.75±0.21	105.29±0.10
I-Propanol	19.74±0.96	18.43±0.14	14.73±0.66	12.61±0.36	2.32±0.29
Isopropyl alcohol	56.67±0.59	51.46±0.44	24.14±0，07	8.73±0.71	3.40±0.49
Glycerol	100.09±0.44	101.51±0.13	101.40±0.59	94.26±0.43	95.20±0.66
Butanol	37.93±0.64	41.60±0.09	85.04±0.33	97.78±0.22	91.01±0.32
Acetonitrile	11.26±0.68	6.06±0.95	4.22±0.84	0.47±0.18	1.97±0.49
Acetone	59.53±0.14	49.83±0.28	12.85±0.17	11.47±0.95	1.58±0.41
Ethyl acetate	101.30±0.18	95.16±0.29	94.36±0.16	96.15±0.38	82.65±0.85

Table S5 Information of β-glucosidase (EC#3.2.1.21) divided into families obtained from CAZy database[a]

GH family	Clan	3D structure status	Catalytic mechanism	Catalytic nucleophile/ base[b]	Catalytic proton donor[b]	Template	Number of potential subsites
GH1	GH-A	$(\beta/\alpha)_8$	Retaining	Glu	Glu[c]		2
GH2	GH-A	$(\beta/\alpha)_8$	Retaining	Glu	Glu		2
**GH3**	**–**	**–**	**Retaining**	**Asp**	**Glu[d]**	**3U48, 5K6M**	**2**
GH5	GH-A	$(\beta/\alpha)_8$	Retaining	Glu	Glu		2
**GH9**	**–**	$\boldsymbol{(\alpha/\alpha)_6}$	**Inverting**	**Asp**	**Glu**	**3X17, 1JS4**	**2**
GH30	GH-A	$(\beta/\alpha)_8$	Retaining	Glu	Glu[e]		2
GH39	GH-A	$(\beta/\alpha)_8$	Retaining	Glu	Glu		2
**GH116**	**GH-O**	$\boldsymbol{(\alpha/\alpha)_6}$	**Retaining**	**Glu**	**Asp**	**5BX5**	**2**
**UC[f]**	–	–	–	–	–	–	–

The families selected for alignment are shown in **bold**.
[a] Information on β-glucosidase (EC#3.2.1.21) is divided into families obtained from CAZy database.
[b] Results were measured from the experimental test.
[c] Glu (experimental); absent in plant myrosinases.
[d] Glu for hydrolases (experimental); histidine for phosphorylases (experimental).
[e] Glu(inferred).
[f] unclassified

Table S6 Sequences of oligonucleotides used for site-directed mutagenesis.

Target sites	Oligonucleotide sequences[a]
S28T	5' CCTAACCATTATCAAAATTATACTTGGTGCAATTTTCAAAATG 3'
	5' CATTTTGAAAATTGCACCAAGTATAATTTTGATAATGGTTAGG 3'
L115N	5' CAAAAAAATAGGATTAATAAACAGAAAATATTTTTTTGAGTGG 5'
	5' CCACTCAAAAAAATATTTTCTGTTTATTAATCCTATTTTTTG 3'
Y37H	5' TTCAAAATGGAGATCACCCTTTTATTGATGGA 3'
	5' TCCATCAATAAAAGGGTGATCTCCATTTTGAA 3'
D44E	5' TATTGATGGAATAGAAATAAACCTAATT 3'
	5' AATTAGGTTTATTTCTATTCCATCAATA 3'
R91G	5' AGTTTATGATTATTTAGGCTTGGAT 3'
	5' ATCCAAGCCTAAATAATCATAAACT 3'
Y82S	5' GTGAAAGTGAAAGAGATTCTATAAAAAGAGTTTATG 3'
	5' CATAAACTCTTTTTATAGAATCTCTTTCACTTTCAC 3'
W122G	5' CTGAGAAAATATTTTTTTGAGGGGAGTAGCCAAATGACTTATG 3'
	5' CATAAGTCATTTGGCTACTCCCCTCAAAAAAATATTTTCTCAG 3'
D44G	5' CCTTTTATTGATGGAATAGGTATAAACCTAATTTATCAGG 3'
	5' CCTTTTATTGATGGAATAGGTATAAACCTAATTTATCAGG 3'

[a]Nucleotide changes are underlined.

Supplementary figure captions

Fig. S1 A Functional screening of second round random mutant library. Hydrolyzing zones produced by *E.coli* strains harbored positive β-glucosidase genes on agar plates, containing ampicillin, esculin hydrate, and ferric ammonium citrate. (a) M2, *E.coli* BL21 (DE3) pLysS contained Q25L/S28T/L115Q/K117N/M148K substitutions. (b) M6, *E.coli* BL21 (DE3) pLysS contained S28P/I57K/Y82S/W112G/E154G substitutions. (c) M20, *E.coli* BL21 (DE3) pLysS contained Y37H/D44E/F68L/I70M/R91G substitutions. **B** Screening of crude enzyme solution of mutant library. White square is as control and it shows the generate amount of ρNP. Black square indicates that remained ρNP amount after the enzyme has been treated at 50 ℃ for 2 h. The experiments were repeated three times.

Fig. S2 SDS-PAGE analysis of the three purified mutant β-glucosidase. Lane 1: protein molecular weight ladder on SDS-PAGE; Lane 2: crude extract of the control *E.coli*; Lane 3: crude extract of the *E.coli*, harboring the expression plasmid with the *bgl1D*; Lane 4-10: purified recombinant Bgl1D, Bgl1D58, Bgl1D 94, Bgl1D 47, Bgl1D2 and Bgl1D6, Bgl1D20 protein.

Fig. S3 Enzymatic properties of β-glucosidases of wild type and mutants using *ρ*NPG as the substrate. **a** Effects of pH on enzyme activity. The enzyme activities were measured at 37 ℃ and pH of 3.0–12.0 in 0.1 M of buffer (pH 3.0–8.0, Na_2HPO_4–citric acid buffer; pH 8.6-10.6, glycine–NaOH buffer; pH 10.9–12.0, Na_2HPO_4–NaOH buffer). **b** Effect of pH on enzyme stability. The enzyme was mixed with 0.1 M of buffers at pH 3.0–12.0 and incubated at 4 ℃ for 24 h. **c** Effects of temperature on the enzyme activity. The enzyme activities were measured at 20 ℃ to 80 ℃ and pH of 10.0. **d** Effects of temperature on the enzyme stability. The enzyme was incubated at 20 ℃ to 80 ℃ for 1 h.

Fig. S4 Determination of glucose/cello tolerance of the heat-resistant enzyme Bgl1D187.

Fig. S5 The transglycosylation HPLC analysis of thermostable mutant enzyme Bgl1D187. **a** Donor is glucose and acceptors is *n*-propanol. Enzyme reaction system of upper figures without thermostable mutant Bgl1D187 as control group. Lower figures added with enzyme as experimental group. **b** Donor is glucose and acceptors is butanol. **c** Donor is cellobiose and acceptor is n-propanol. **d** Donor is glucose and acceptor is butanol.

Figure S1

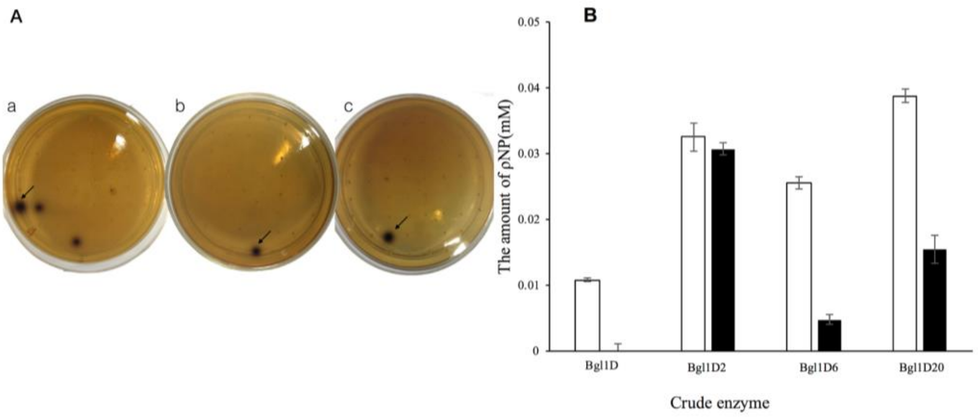
A
a
b
c
B
The amount of ρNP(mM)
0.05
0.04
0.03
0.02
0.01
0
Bgl1D
Bgl1D2
Bgl1D6
Bgl1D20
Crude enzyme

Figure S2

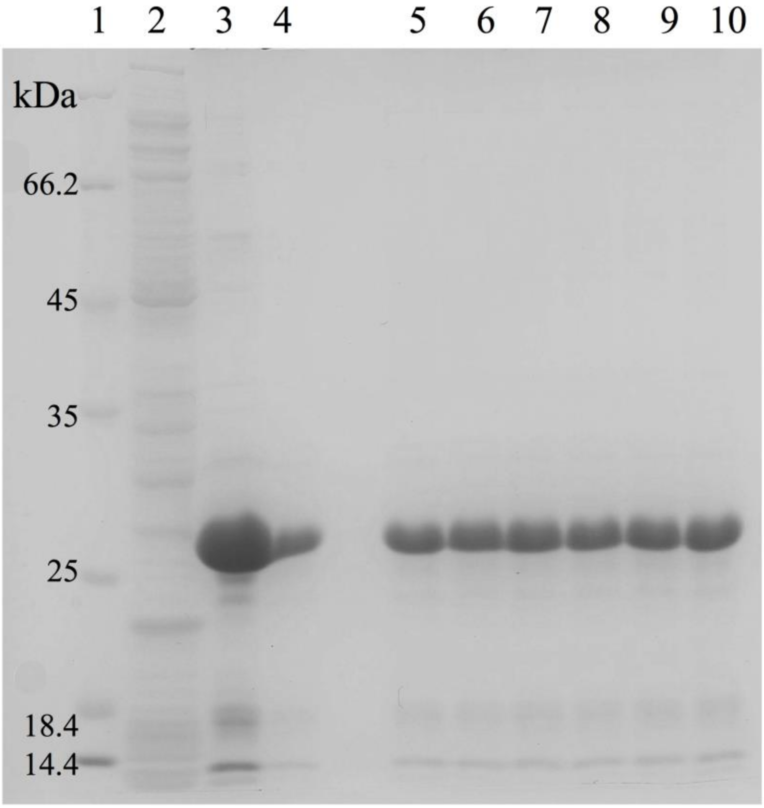

Figure S3

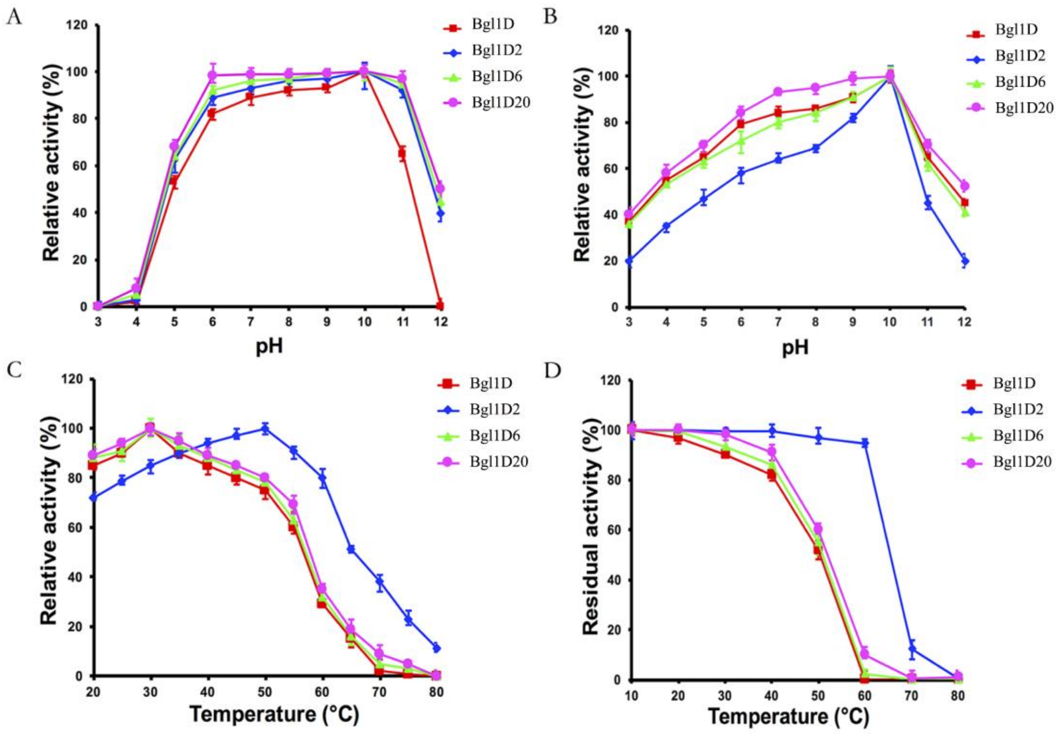
A
B
C
D
Bgl1D
Bgl1D2
Bgl1D6
Bgl1D20
Relative activity (%)
Residual activity (%)
pH
Temperature (°C)
0
20
40
60
80
100
120
3
4
5
6
7
8
9
10
11
12
10
20
30
40
50
60
70
80

Figure S4

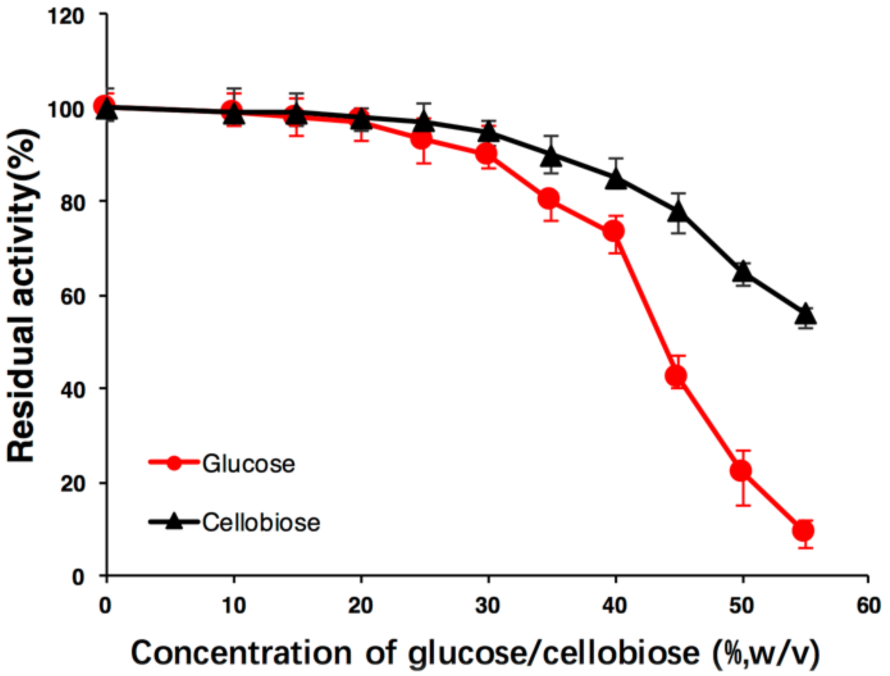
Residual activity(%)
120
100
80
60
40
20
0
Glucose
Cellobiose
0
10
20
30
40
50
60
Concentration of glucose/cellobiose (%,w/v)

Figure S5

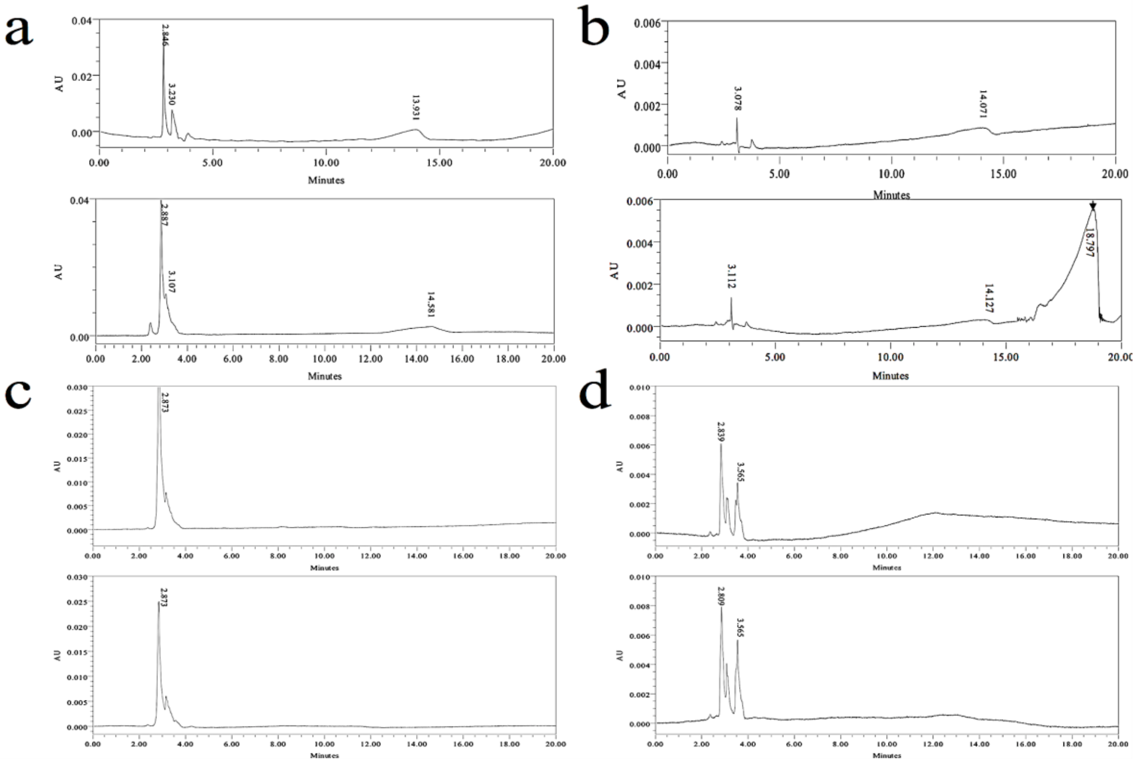
a
b
c
d
AU
Minutes
2.846
3.230
13.931
2.887
3.107
14.581
3.078
14.071
3.112
14.127
18.797
2.873
2.839
3.565
2.809
